# Conversación pública sobre vacunas en la pandemia de covid-19 en Argentina, 2021-2022

**DOI:** 10.18294/sc.2024.4580

**Published:** 2024-02-21

**Authors:** Flavia Demonte, Daniela Paola Bruno, Leandro Simón Lozano, Maria Florencia Mena, Andrés Martín Pereira

**Affiliations:** 1 Posdoctora en Ciencias Sociales. Investigadora Adjunta, Consejo Nacional de Investigaciones Científicas y Técnicas, con sede en Escuela Interdisciplinaria de Altos Estudios Sociales, Universidad Nacional de San Martín, Buenos Aires, Argentina. fdemonte@unsam.edu.ar Consejo Nacional de Investigaciones Científicas y Técnicas Escuela Interdisciplinaria de Altos Estudios Sociales Facultad de Ciencias Sociales Consejo Nacional de Investigaciones Científicas y Técnicas Buenos Aires Argentina fdemonte@unsam.edu.ar; 2 Posdoctora en Ciencias Sociales. Investigadora, Facultad de Periodismo y Comunicación Social, Universidad Nacional de la Plata. Investigadora, Instituto de Investigaciones Gino Germani, Facultad de Ciencias Sociales, Universidad de Buenos Aires, Ciudad Autónoma de Buenos Aires, Argentina. danielapaolabruno@gmail.com Universidad de Buenos Aires Instituto de Investigaciones Gino Germani Facultad de Ciencias Sociales Universidad de Buenos Aires Ciudad Autónoma de Buenos Aires Argentina danielapaolabruno@gmail.com; Facultad de Periodismo y Comunicación Social Universidad Nacional de la Plata; 3 Licenciado en Comunicación Social. Epidemiólogo, Dirección de VIH, ITS y HV. Epidemiólogo, Hospital Cuenca Alta Néstor Kirchner. Coordinador docente, Residencia de Epidemiología, Ministerio de Salud de la Provincia de Buenos Aires, Buenos Aires, Argentina. leandroslozano@gmail.com Ministerio de Salud de la Provincia de Buenos Aires Residencia de Epidemiología Ministerio de Salud de la Provincia de Buenos Aires Buenos Aires Argentina leandroslozano@gmail.com; Dirección de VIH, ITS y HV; 4 Licenciada en Ciencias Antropológicas. Becaria doctoral, Consejo Nacional de Investigaciones Científicas y Técnicas, Ciudad Autónoma de Buenos Aires, Argentina. mflorenciamena@gmail.com Consejo Nacional de Investigaciones Científicas y Técnicas Consejo Nacional de Investigaciones Científicas y Técnicas Ciudad Autónoma de Buenos Aires Argentina mflorenciamena@gmail.com; 5 Especialista en Epidemiología. Profesor en Ciencias Antropológicas. Docente adjunto, Universidad Nacional de Tres de Febrero. Antropólogo, CeSAC N°24, Ministerio de Salud del Gobierno de la Ciudad de Buenos Aires, Ciudad Autónoma de Buenos Aires, Argentina. andres.m.pereira@gmail.com Universidad Nacional de Tres de Febrero Universidad Nacional de Tres de Febrero Ciudad Autónoma de Buenos Aires Argentina andres.m.pereira@gmail.com; CeSAC N°24 Ministerio de Salud del Gobierno de la Ciudad de Buenos Aires

**Keywords:** Vacunación, Comunicación, Gobierno, Medios de Comunicación, Red Social, Argentina, Vaccination, Communication, Government, Communications Media, Social Networking, Argentina

## Abstract

Ante la caída de las coberturas vacunales y la circulación informativa sobre salud, las conversaciones en el entorno público/mediático digital constituyen un ámbito de estudio relevante para el campo de la comunicación en salud. A través de un estudio cualitativo, basado en el análisis de publicaciones del gobierno, la prensa digital y las redes sociales, caracterizamos la conversación pública sobre vacunas -en términos de temas, momentos, ejes y encuadres en Argentina en el período 2020-2021- signada por el debate sobre las vacunas covid-19. Los resultados muestran que la conversación pública se centralizó en la vacunación contra el covid-19, se estructuró en dos momentos diferenciados (producción de vacunas y campaña de vacunación) y bajo encuadres morales sustentados en la vacunación como práctica de cuidado y la ciencia como voz autorizada. En simultáneo, las dudas sobre la seguridad y eficacia de las vacunas estructuraron argumentos de reticencia vacunal, que entendemos como parte de prácticas extendidas, asociadas con las desconfianzas hacia las instituciones y reinterpretaciones del conocimiento científico y del cuidado.

## Abstract (en)

In the face of declining vaccination coverage and the dissemination of health-related information, conversations in the public/mediatic digital sphere constitute a relevant study area for the field of health communication. Through a qualitative study based on the analysis of government publications, digital press, and social media, we characterize the public conversation on vaccines - in terms of topics, moments, axes, and framings in Argentina during the 2020-2021 period - marked by the debate on covid-19 vaccines. The results show that public conversation focused on covid-19 vaccination, structured in two distinct moments (vaccine production and vaccination campaign), and under moral framings grounded in vaccination as a care practice and science as an authoritative voice. Simultaneously, doubts about the safety and efficacy of vaccines shaped arguments of vaccine hesitancy, which we understand as part of extended practices associated with distrust towards institutions and reinterpretations of scientific knowledge and care.

## INTRODUCCIÓN

Si bien la interrupción de los servicios de vacunación por la pandemia de covid-19 impactó en las coberturas a nivel mundial, ya desde 2010 las tasas de cobertura de vacunas vitales se habían mantenido en el 86% cuando los especialistas estimaban que se necesitaba una cobertura del 95% para considerarla óptima^1^. En 2019, Argentina presentaba coberturas casi óptimas, aunque existían diferencias entre jurisdicciones y vacunas, y se observaba una disminución en la cobertura de la mayoría de ellas. En el primer año de la pandemia, en Argentina se registró́ un descenso promedio de 10 puntos respecto de 2019^2^, por lo que el Ministerio de Salud de la Nación alertó sobre su profundización a causa del Aislamiento Social Preventivo y Obligatorio (ASPO) y definió la vacunación como acción prioritaria.

La caída de la vacunación es un fenómeno complejo que depende del contexto, el momento, el lugar, el tipo vacuna y la interacción de determinantes sociales diversos que influyen en la decisión de vacunar. Antes de la pandemia, ya la Organización Mundial de la Salud había alertado sobre el crecimiento de la desconfianza hacia las vacunas, identificándola como una de las diez amenazas más importantes a la salud pública global^3^.

Los estudios argentinos recientes sobre conocimiento, aceptación y reticencia vacunal con enfoques cuantitativos^4^^,^^5^ parten del supuesto de que la reticencia es resultado del desconocimiento de información biomédica, simplificando el complejo proceso de atribución de significado y su vínculo con las prácticas de cuidado de la salud. Pero los estudios cualitativos^6^^,^^7^ evidencian explicaciones más complejas que comprenden que las significaciones se gestan en el marco de grupos sociales, atravesados por climas de época, contextos específicos y culturas de vacunación.

En un estudio sobre los conocimientos que tienen los padres y las madres de la Ciudad Autónoma de Buenos Aires (CABA) sobre vacunas y sobre los argumentos a los estas personas recurren para expresar aceptación, desconfianza o renuencia a la vacunación de sus hijos e hijas en el contexto del desarrollo de vacunas covid-19^8^, la población estudiada reconoció la efectividad de la vacunación como práctica preventiva, pero expresó dudas y temores sobre la seguridad y eficacia de vacunas específicas (covid-19, virus del papiloma humano y antigripal). Algunas personas pusieron en duda la necesidad de aplicar vacunas para enfermedades erradicadas y cuestionaron la obligatoriedad de la vacunación, argumentando el respeto a la autonomía en las decisiones sobre la propia salud, sobre todo en personas informadas con posiciones críticas sobre el modelo biomédico.

Asumiendo que las personas buscan información sobre el cuidado de la salud, más allá del entorno personal en el actual contexto hipermediatizado, en la última década se incrementaron y diversificaron notablemente los estudios sobre: la información circulante en los medios de comunicación y la incidencia que las *fake news* y *fake sciences* tienen en la significación social de las vacunas, en particular las de covid-19^9^^,^^10^^,^^11^^,^^12^; el modo en que la plataformización, algoritmización y datificación inciden en el acceso a información, incrementando la polarización social y abonando la desconfianza hacia las vacunas^13^; el tratamiento periodístico de la vacuna y la campaña de vacunación covid-19^14^; la comunicación gubernamental sobre covid-19 en redes sociales^15^^,^^16^; la caracterización de los denominados grupos antivacunas y sus argumentos en la conversación digital en redes sociales^17^^,^^18^^,^^19^^,^^20^; la narrativa de los organismos encargados de la aprobación de vacunas covid-19^21^ y el consumo informativo sobre covid-19 en redes sociales^22^^,^^23^.

Considerando la incidencia que la mediatización y la opinión pública tienen en las actitudes hacia la vacunación, este artículo tiene como objetivo caracterizar la conversación pública sobre vacunas (temas, momentos, ejes y encuadres) en Argentina durante 2020-2021, periodo signado por el debate sobre las vacunas covid-19. Desde la perspectiva teórica que anima el artículo, se asume que el carácter público de un problema es la atención que este suscita, especialmente en el registro de la acción pública, lo que comprende no solo a los poderes públicos, sino a toda acción articulada en el espacio público, movilizando alguna referencia al bien común^24^. Por tanto, circunscribe el análisis de la conversación pública digital a las publicaciones en medios de comunicación gubernamental de nivel nacional, al tratamiento otorgado al tema por parte de la prensa digital y a las publicaciones e interacciones sobre la temática en las cuentas de Facebook, Twitter y YouTube de asociaciones científicas ligadas a la vacunación y colectivos sociales movilizados en torno a las vacunas, actores centrales en el debate en torno al tema de conversación.

## COORDENADAS TEÓRICO-METODOLÓGICAS

Se realizó un estudio exploratorio-descriptivo a partir de un marco conceptual que puso en diálogo aportes de la sociología de los problemas públicos^25^, la teoría del *framing*^26^^,^^27^^,^^28^ y la semiótica de las mediatizaciones^29^^,^^30^^,^^31^. Específicamente, de la sociología de los problemas públicos, para la definición del abordaje analítico del problema, se apeló a la noción de problema público de Joseph Gusfield^25^, en tanto constructo dinámico y conflictivo, haciendo foco en las situaciones de intercambio simbólico y discursivo en las que confluyen y/o colisionan distintas respuestas a preguntas que definen la configuración retórica y dramática del problema. También se consideraron otras tres nociones dentro del sistema conceptual referenciado: propietarios (del problema), responsables causales y políticos (del problema) y estabilización (del problema). Dado que los problemas son el resultado de procesos de tematización (o publicización) y movilización e interpelación de entidades colectivas (públicos) dentro de un campo de interacción y disputa (al que el autor denominó arena pública)^25^, y atendiendo a que para lograr atención y credibilidad en la arena público-mediática los actores sociales echan mano a sistemas categoriales, para el análisis de dicha caracterización y justificación se recurrió a la teoría del *framing*, específicamente al concepto de marco (*frame*) y enmarcamiento o encuadre (*framing*) de Robert Entman^26^. En términos operativos, recurrimos específicamente a los encuadres genéricos e indicadores propuestos por Holli Semetko y Patti Valkenburg^28^: juicio moral, conflicto, atribución de responsabilidad, interés humano, consecuencias económicas. Finalmente, de la semiótica de las mediatizaciones tomamos la caracterización del sistema mediático realizada por Mario Carlón^29^, en la que los medios de comunicación tradicionales conviven e interactúan con los medios personales en un nuevo ecosistema de medios y plataformas con base en Internet que se expande vertiginosamente y se caracteriza por la digitalización, la convergencia, la interactividad y la disponibilidad de contenidos en línea, reclamando lecturas más atentas a la circulación y a los flujos de sentido no lineales que se despliegan en las redes sociales^29^^,^^31^. Además, para el análisis operativo de las publicaciones, se consideró en términos amplios la tesis básica del análisis de las mediatizaciones propuesta por José Luis Fernández^31^, que postula que en los textos/mensajes que se intercambian se encuentran marcas que se constituyen en huellas de los dispositivos técnicos con los que están construidos y distribuidos, de las clasificaciones genérico-estilísticas en las que se inscriben, y de las propuestas de prácticas comunicacionales y relaciones con usos sociales; y de los actores que intervienen. También se prestó especial atención a la secuencia metodológica propuesta por el autor para el análisis de la dimensión temática, retórica y enunciativa de las publicaciones.

**Figura 1:**
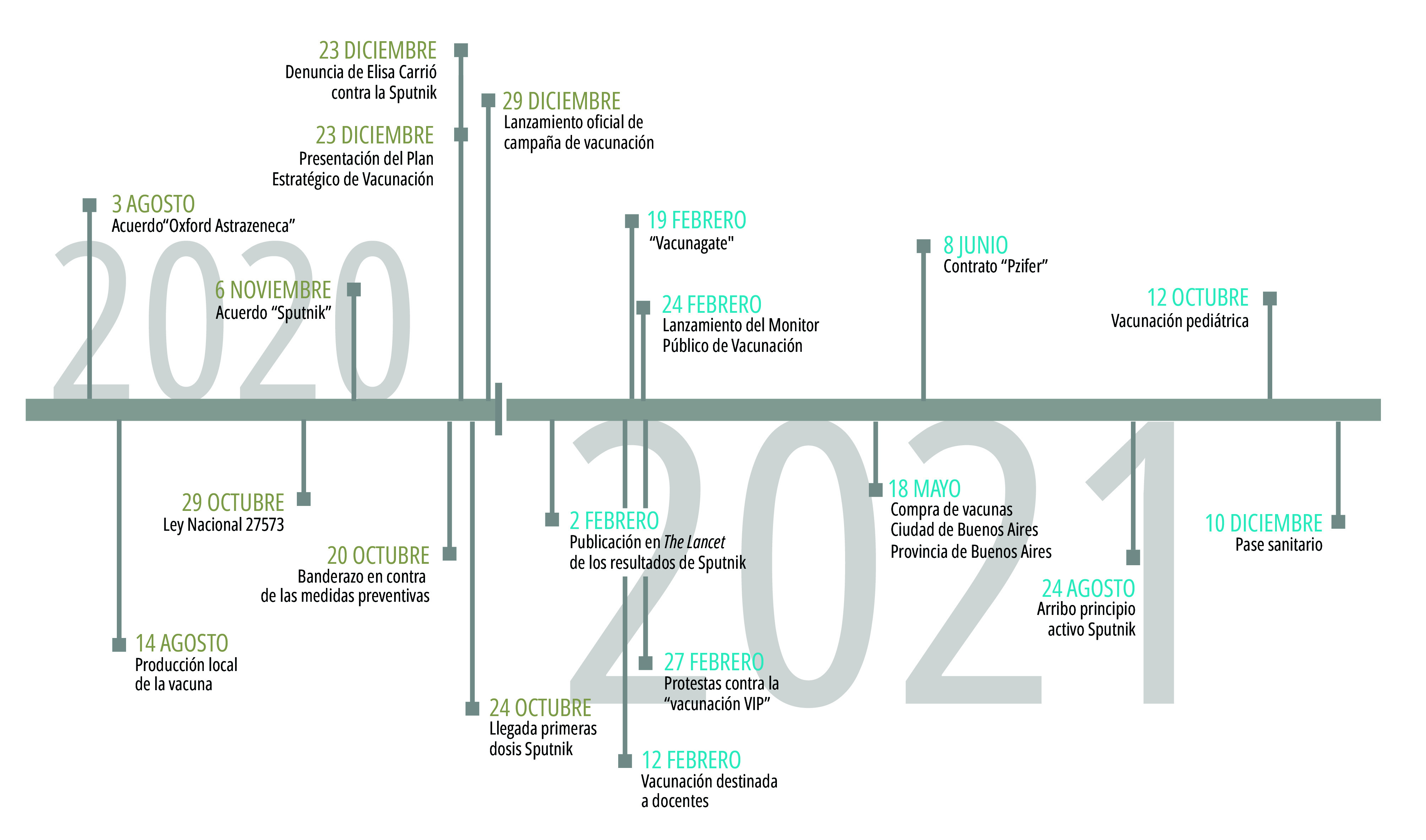
Hitos relacionados con la vacunación y las vacunas de covid-19. Argentina, agosto 2020- diciembre 2021.

Metodológicamente, la búsqueda de información según fuente (prensa, gobierno, cuentas relevantes en redes sociales) se realizó contemplando 19 hitos relevantes, relativos a la vacunación y las vacunas, identificados a partir de un análisis preliminar de la actividad oficial del gobierno nacional informada en el sitio web de Presidencia, en diálogo con la cobertura periodística a nivel nacional y una primera exploración temática de las publicaciones en redes sociales (Figura 1).

Fuente: Elaboración propia. Notas: Elisa Carrió es fundadora del partido Coalición Cívica ARI y fue diputada nacional por el Chaco y por la Ciudad de Buenos Aires. Al momento de realizar la denuncia, integraba las fuerzas de la oposición al gobierno de Alberto Fernández. “Vacunagate” es una de las denominaciones del “Vacunatorio VIP

Para el relevamiento de las publicaciones en redes sociales (Twitter, Facebook y YouTube); en la prensa digital (*La Nación*, *Clarín*, *Infobae*, *Página/12*) y en los mensajes del gobierno nacional (página web de Presidencia y del ex Ministerio de Salud; y las cuentas de Twitter del presidente y del ministro y la ministra de Salud durante el período) los criterios de búsqueda fueron: a) temáticos (covid-19, vacunas); b) geográficos (Argentina); c) temporales (2020 y 2021, circunscriptos a los siete días posteriores de cada uno de los 19 hitos). Adicionalmente, en el caso de las redes sociales, específicamente YouTube y Twitter, además de los criterios antedichos se prestó especial atención a las publicaciones con mayor número de interacciones y réplicas en cuentas de personalidades públicas, instituciones y colectivos sociales con incidencia en el debate público sobre vacunas. En el caso de Facebook, luego de aplicados los criterios generales de búsqueda, se seleccionaron intencionalmente las publicaciones de personas y grupos públicos competentes o activistas en la temática, como sociedades científicas, personas o colectivos sociales críticos a la vacunación.

**Figura 2:**
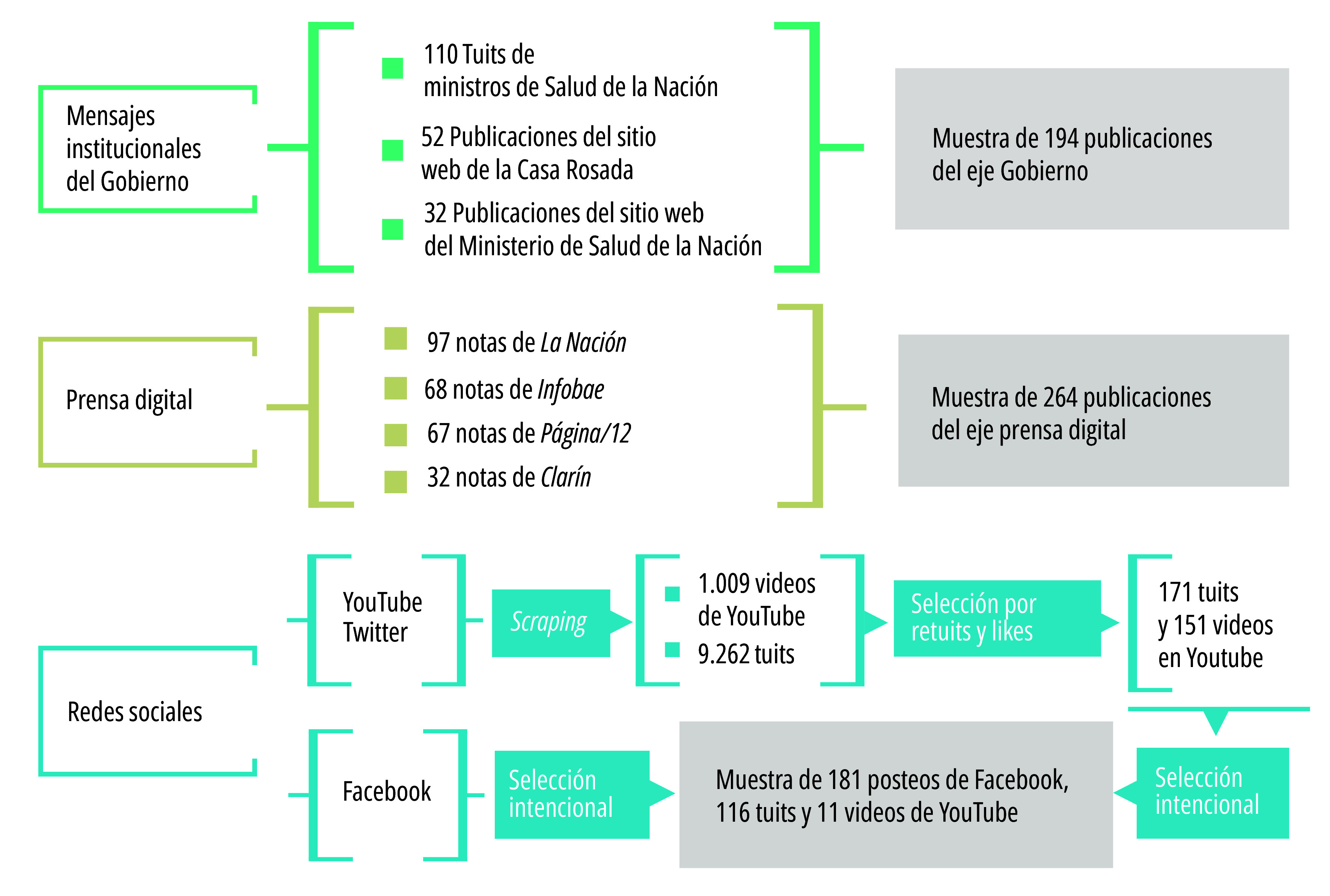
Descripción de la muestra de publicaciones sobre la vacunación y las vacunas covid-19, según actor y/o plataforma (n=766). Argentina, agosto 202 - diciembre 2021.

La muestra quedó conformada por 766 publicaciones, distribuidas en 194 publicaciones del gobierno, 264 noticias de prensa digital y 308 posteos y videos de redes sociales (Figura 2).

Fuente: Elaboración propia.

Configurada la muestra, se confeccionó un libro de categorías (Tabla 1) y se procedió a su codificación y análisis temático asistido con soporte informático (Atlas.TI).

**Tabla 1:** Categorías temáticas y definiciones conceptuales para el análisis de las publicaciones sobre la vacunación y las vacunas de covid-19. Argentina, agosto 2020 - diciembre 2021.

Categorías temáticas	Definiciones conceptuales
Gestión	Enunciados referidos a la gestión de la pandemia y la atención a la población; a fundamentos, principios y valores de la política general y sanitaria; a medidas, instrumentos y políticas sociales, socio laborales, económicas, educativas para el manejo de la pandemia.
Controversias	Enunciados referidos a aspectos controversiales vinculados con seguridad de Sputnik V; obligatoriedad de vacuna covid-19; vacunación pediátrica; inicio de clases presenciales.
Vacuna	Enunciados referidos a la vacuna covid-19, su investigación y desarrollo, producción y compra o adquisición hasta su disponibilidad para ser distribuida. Incluyó referencias a seguridad y eficacia de la vacuna y sus diferentes marcas.
Campaña de vacunación	Enunciados referidos al desarrollo de la campaña de vacunación como política sanitaria, incluyendo la coordinación para plan estratégico de vacunación, logística, distribución, preservación, criterios de priorización por grupos poblacionales, esquema de vacunación.
Argumentos	Enunciados argumentativos a favor o en contra de la vacunación.
Epidemiología	Enunciados referidos a dimensiones epidemiológicas no contempladas en otras categorías, como información epidemiológica en relación con la pandemia; referencias a conducta social y su impacto en la salud a nivel poblacional; medidas sanitarias de contención o control de casos; mensajes preventivos en el marco de la campaña de vacunación.
Escándalos/affaire	Enunciados referidos a momentos disruptivos de alto impacto en la arena pública.
Evaluación moral del comportamiento ciudadano	Enunciados referidos a valoraciones o juicios morales sobre el comportamiento ciudadano.
Vacunas calendario	Enunciados referidos a otras vacunas del calendario nacional.
Enseñanzas pandémicas	Enunciados relacionados con la dimensión pedagógica de la pandemia como oportunidad de aprendizaje, campaña, vacunación.
Salud (pregestión expresidente Alberto Fernández)	Enunciados referidos al “objeto salud” en relación con políticas públicas previas a la “gestión Alberto Fernández”.
Antivacunas, movimientos	Enunciados que caractericen y refieran a actores que participan activamente en contra de la vacunación.

Fuente: Elaboración propia.

En una primera instancia se cuantificaron las menciones para dar cuenta de la relevancia de cada una de las categorías, fragmentando los textos en unidades de codificación para su agrupamiento en las categorías previstas. Luego, se establecieron vínculos entre ellas para identificar las de mayor convergencia y conflicto y, a partir de la interpretación de sus relaciones y estructuras argumentativas, se elaboró una síntesis analítica de momentos y ejes de la conversación. Finalmente, se realizó una aproximación retórico enunciativa para identificar los encuadres de la conversación pública, las huellas de los dispositivos técnicos involucrados en la construcción y distribución de los contenidos, el tipo de clasificaciones genérico-estilísticas dominantes, y la trama actoral y discursiva reconocible en el conjunto de textos analizados, según la secuencia metodológica propuesta por los autores de referencia^29^. En los apartados siguientes, con el propósito exclusivo de ejemplificar afirmaciones que surgieron del análisis, se destacan eslóganes de la comunicación gubernamental, y se citan fragmentos de las declaraciones de las autoridades, de las noticias de la prensa digital y posteos de cuentas de las redes sociales relevantes, seleccionados intencionalmente y sin pretensión de exhaustividad. En estos últimos casos y por tratarse de publicaciones públicas, no se consideró necesario anonimizarlos. Por la misma razón, no se requirió de la evaluación de un comité de ética en investigación en salud.

## TÓPICOS FRECUENTES EN LAS PUBLICACIONES

**Figura 3:**
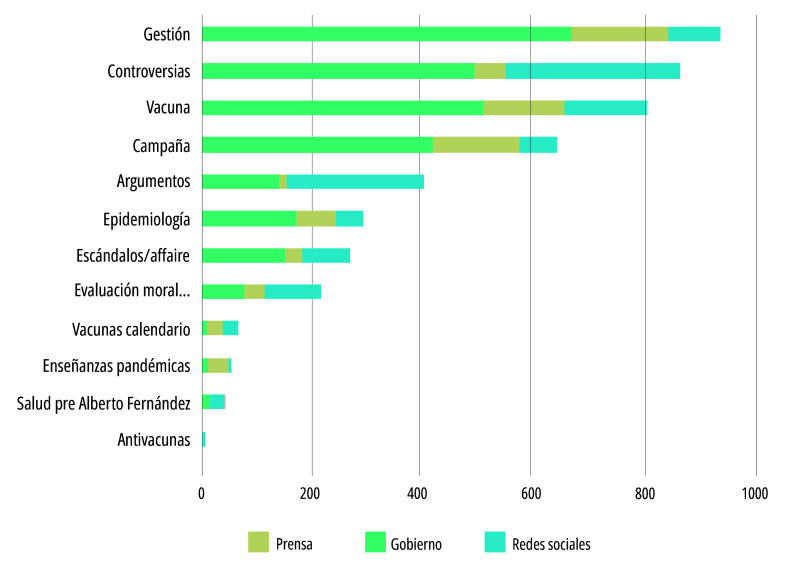
Frecuencia de categorías temáticas sobre la vacunación y las vacunas covid-19, según actor y/o plataforma (n=4.647 citas en 766 publicaciones reveladas). Argentina, agosto 2020 - diciembre 2021.

Como se aclaró en el apartado anterior, el análisis de los problemas públicos se interesa particularmente por las tematizaciones que mayor atención, credibilidad o disputa concitan acerca de un asunto, pues son estas las que dan forma a un problema en la arena público-mediática^25^. Esto nos llevó, inicialmente, a identificar cuáles habían sido los temas que mayor atención habían concitado en las publicaciones del gobierno, la prensa y los actores sociales interesados en la vacunación. Teniendo esto en cuenta, en la Figura 3 pueden observarse las categorías temáticas que más menciones tuvieron en el conjunto de publicaciones analizadas. Dentro de las primeras cinco, la categoría *gestión* de la pandemia ocupó el primer lugar, por el número de menciones que tuvo tanto en la prensa digital como en la comunicación gubernamental. En segundo lugar, la categoría *controversias*, con un reparto equitativo entre los diferentes actores y plataformas indica la atención y/o cobertura que tuvieron diversos asuntos controversiales (la seguridad de la vacuna Sputnik V, la obligatoriedad de la vacunación contra el covid-19, el inicio de las clases presenciales en la Ciudad Autónoma de Buenos Aires, la vacunación pediátrica), con protagonismo en redes sociales y en la prensa digital. La tercera categoría, *vacuna covid-19*, concentró mayor atención en la prensa digital y en la comunicación gubernamental, al igual que *campaña* (de vacunación) covid-19 en el cuarto lugar, que fue relevante en la prensa digital y en la comunicación gubernamental, pero no concentró atención en redes sociales. A propósito de esta cuestión, resultó llamativo el bajo número de menciones que tuvieron las vacunas del calendario nacional. En el conjunto de actores y plataformas estudiados, estas fueron eclipsadas o postergadas por la centralidad que adquirió la vacuna covid-19 y su campaña de vacunación. Por último, los argumentos favorables o críticos a la vacunación, en el quinto lugar, fueron más frecuentes en redes sociales y en prensa digital, indicando el carácter polémico que allí tuvo la vacunación contra el COVID-19 como práctica de cuidado, sin que esto haya justificado un tratamiento semejante en la comunicación gubernamental.

Fuente: Elaboración propia.

Los tópicos con menciones más frecuentes no se distribuyeron homogéneamente en los diferentes actores y plataformas, ni a lo largo del periodo. Esto configuró dos momentos de la conversación sobre la vacunación en general y la vacuna covid-19 en particular: el primero, en torno a la producción de vacunas covid-19 y, el segundo, sobre el diseño y el desarrollo de la campaña de vacunación covid-19.

### El desarrollo de vacunas covid-19

A inicios del periodo estudiado, el desarrollo de vacunas covid-19 fue objeto de atención privilegiada en la conversación pública^22^, aunque con matices. Estos matices fueron reconocidos a partir del análisis de la circulación hipermediática del sentido, cuyo punto de partida fue generalmente la voz gubernamental, luego amplificada y, en algunos casos, cuestionada por la prensa digital. En la comunicación gubernamental se priorizó el esfuerzo del gobierno por firmar contratos con todos los laboratorios bajo el imperativo del no lucro, guiando la celebración de los acuerdos y el propósito de un acceso oportuno y eficiente a precios razonables. Además, el gobierno enfatizó el orgullo por la calidad del trabajo científico nacional en su proceso de producción y aprobación. En la prensa digital y las redes sociales ocurrió algo similar, aunque con mayor énfasis en los debates sobre la seguridad de las vacunas y la legitimidad de los organismos certificadores. La negociación con laboratorios, las decisiones de producción local y/o transferencia tecnológica y las compras se asociaron con decisiones de posicionamiento geopolítico antes que con fundamentos científicos y/o técnicos. Las complicaciones para garantizar la disponibilidad de las dosis y la logística para distribuirlas fueron interpretadas como efectos de esos posicionamientos. Hacia fines de 2020, adquirió centralidad en la prensa digital y las redes sociales la controversia acerca de la seguridad de la vacuna Sputnik V y las prerrogativas que el gobierno habría concedido al gobierno ruso en detrimento de otras alternativas^8^^,^^22^. El presidente de la Nación Alberto Fernández enfatizó la seguridad de la vacuna garantizada por el Instituto Gamaleya y la Administración Nacional de Medicamentos, Alimentos y Tecnología Médica, calificando las críticas de la oposición y de la prensa como malintencionadas, extemporáneas y/o irracionales. Clausurado el debate sobre la vacuna rusa luego de la publicación de un artículo favorable en la revista *The Lancet*, el presidente Alberto Fernández declaró:

Nosotros seguimos negociando con todos, lo que pasa es que, en la locura de algunos, así como me acusaron de envenenar gente [...] Ahora, resulta ser que el veneno fue la vacuna más efectiva de todas las que se produjeron en el mundo y ahí ya no dijeron más nada. (Sitio web de la Presidencia, Prensa, mayo 2021)

A comienzos de 2021, cuando la vacuna rusa llevaba un mes siendo aplicada y la adquisición de vacunas Pfizer se vio frustrada por incompatibilidades con la legislación, parte de la prensa y personas usuarias de las redes sociales lo interpretaron como una maniobra del gobierno para centrar la compra en la Sputnik V. Si bien el gobierno respondió a las críticas que circularon en medios periodísticos y publicaciones de redes sociales de actores públicos, a partir de ese momento la comunicación gubernamental se centró en el reclamo por la vacuna como bien público global, destacando la producción de la vacuna nacional.

### La campaña de vacunación para covid-19

Un segundo momento de la conversación se centró en el desarrollo de la campaña de vacunación covid-19 y las polémicas suscitadas por decisiones gubernamentales sobre su implementación. Al igual que en el primer momento, el gobierno nacional orientó la agenda periodística con excepción del escándalo político conocido como “vacunatorio VIP” en el que la prensa digital y algunas cuentas de las redes sociales centralizaron la discusión. En este contexto, junto a la categoría *vacunación covid-19*, aparecieron en la conversación tópicos incluidos en las categorías *controversias y escándalos/affaires*. Así, apenas iniciada la vacunación, la atención pública se concentró en el escándalo político^22^ conocido como “vacunatorio VIP”. En la prensa digital se observaron críticas a la actitud del entonces ministro, con tendencia a la generalización, incluyendo a todo el gobierno. Se enfatizó en el carácter secreto de las negociaciones y el uso de herramientas de gestión para sostener privilegios. *Página/12*, condenó la situación, pero encontró una justificación en el carácter excepcional del hecho y enfocó la atención en las medidas para salir de esa situación de crisis.

Las redes sociales fueron el escenario para la expresión de la indignación y la convocatoria a la movilización de la ciudadanía por parte de la oposición al partido gobernante, apelando al relato de casos de personas que murieron antes de vacunarse, adjudicando la responsabilidad a los “vacunados vip”.

Es peligroso para nuestra democracia que sectores de la oposición insistan en profundizar los discursos de odio. ¿Es odio lo único que tienen para ofrecerle a la sociedad? https://t.co/tMlXalZOu5 (Twitter, Santiago Cafiero, febrero de 2021, retwits 1.322 - likes 5.905)

**Figura 4:**
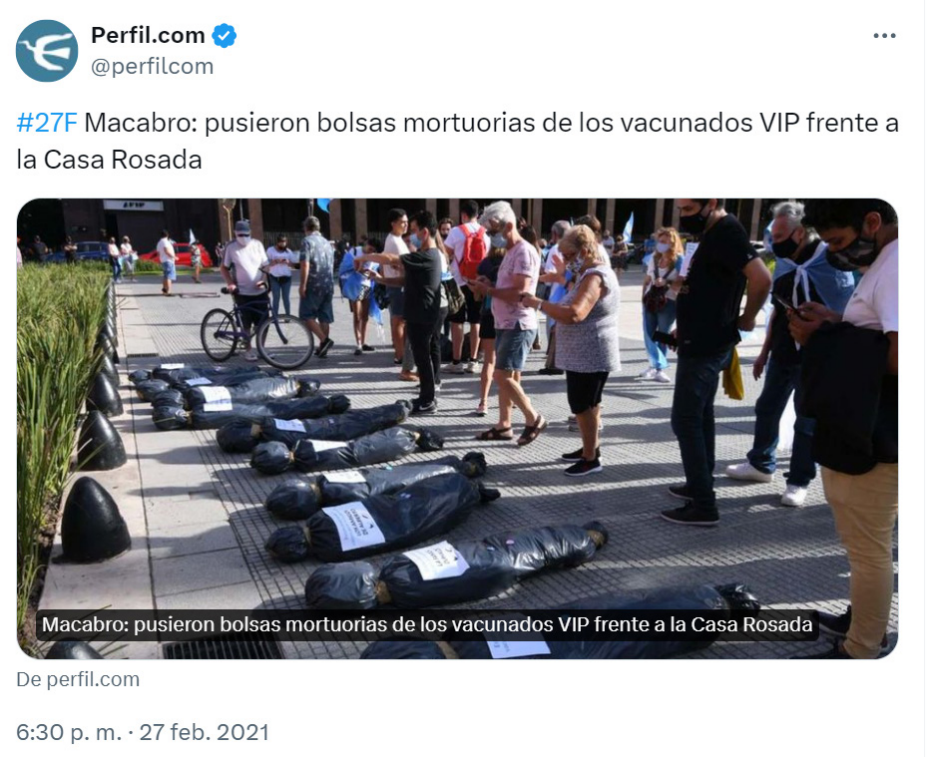
Bolsas mortuorias con nombres de dirigentes políticos depositadas frente a las rejas de la Casa Rosada por las personas que se manifestaron en contra del “Vacunatorio VIP”. Argentina, febrero de 2021.

El posteo de Santiago Cafiero, por entonces jefe de Gabinete de Ministros de la Nación, cita el siguiente tuit del diario *Perfil* (Figura 4):

Fuente: Diario Perfil.

Vivo en un País dónde es más importante una bolsa Asurín que un político que nos afana, nos miente, nos divide, se queda con la vacuna de tus viejos. Despertate boludo. (Twitter, Diego Poggi, febrero de 2021, retuit 5.370 - likes 22.079)

Por su parte, la comunicación gubernamental de la campaña de vacunación covid-19 enfatizó algunos de sus rasgos: por un lado, su carácter federal; por otro, su condición equitativa y proporcional (por el criterio con el que el Estado adquirió y distribuyó las vacunas según densidad poblacional y por grupos priorizados con base en el riesgo de vida y por ser simultánea en todo el país); y, por último, su envergadura sin precedentes (por el esfuerzo logístico y la población a alcanzar). En respuesta a las críticas recibidas a partir del escándalo del “vacunatorio VIP”, en febrero de 2021, se incrementó el énfasis en la equidad distributiva de las vacunas y la transparencia de su gestión con el lanzamiento del “Monitor público de vacunación” con información en línea sobre el operativo nacional, como una demostración de transparencia luego del escándalo que provocó la renuncia del ministro Ginés González García y la designación de Carla Vizotti. “#Ya estamos vacunando”, sintetizó la línea de comunicación del gobierno abocada a informar sobre la campaña, brindando información sobre: dosis adquiridas, recibidas y repartidas; grupos priorizados, esquema de dosis; sus logros (dosis entregadas, personas vacunadas, tasas de mortalidad) y sus obstáculos (escasez de vacunas en un contexto de alta demanda). En la prensa digital favorable al gobierno se observó que no solo se informó cotidianamente sobre la inclusión de la población objetivo, conforme los criterios establecidos en el plan de vacunación, sino también sobre la envergadura sin procedentes de la campaña, acompañando la comunicación gubernamental. Como logros, se puso énfasis en la cantidad de inscriptos y vacunados, las dosis distribuidas y aplicadas; y en el comienzo de la campaña, nombrándolo como día histórico y epopeya, recuperando las palabras del ministro de Salud, como fuente privilegiada de información. Como debilidades o críticas se reconoció el déficit de dosis durante los primeros meses de la campaña, la compra poco diversificada y la falta de transparencia sobre todo a partir del escándalo.

En consonancia con la prensa digital, específicamente en Facebook, se advirtieron explicaciones sobre las modificaciones realizadas en los criterios de inclusión y definición de grupos priorizados, y caracterizaciones de la campaña, los recursos necesarios, los componentes de las vacunas y los logros, provenientes de cuentas de sociedades científicas o ligadas al Consejo Nacional de Investigaciones Científicas y Técnicas (CONICET). Tanto en Twitter como en YouTube, se observaron pocas referencias a la campaña de vacunación, consistentes en mensajes compartidos por portavoces del gobierno. Sobre todo en los inicios de la campaña, se registraron algunos cuestionamientos a los criterios de selección de los grupos priorizados, la lentitud y la eficiencia de la campaña.

Jorge Gilardi: ¿Y a quién van a vacunar? van a vacunar a las UTIs [unidades de terapia intensiva], perfecto, a todos los que estuvieron en las UFUS [unidades febriles de urgencia], perfecto, los que andan en las ambulancias, muy bien. Y aquellos en los laboratorios, bioquímicos y colegas que están trabajando en una primera línea en contacto con el COVID, y yo pregunto ¿y las guardias? ¿y los centros de salud? (YouTube, diciembre de 2020, 382.768 visualizaciones)

En octubre de 2021, la ministra de Salud dio la “bienvenida a los niños y las niñas al plan de vacunación más importante de la historia argentina”, lo que se constituyó en objeto controversial en la prensa digital y en redes sociales, aunque tuvo, de parte de la comunicación gubernamental, un tratamiento técnico. La única publicación oficial referida a la controversia se observó en la cuenta de Twitter de la ministra quien compartió una publicación de Ernesto Resnik, en la que el biólogo compartía un artículo de la revista *Science*, respaldando la vacunación pediátrica. Sobre este asunto, se observaron posteos en redes sociales por parte de personas usuarias contrarias a la vacunación contra el covid-19.

Ni efectivas, ni seguras mintieron y siguen haciéndolo. La realidad viene demostrando que los experimentos no reducen los supuestos contagios, no evitan las formas graves, más aún se asocian a múltiples efectos adversos [...] y encima siguen proponiendo más dosis queriendo imponerlas de manera coercitiva y absolutamente ilegal. ¡Reaccionen, estamos en guerra! Lo que estamos padeciendo es una plena dictadura política sanitaria. ¡Digamos basta! (Facebook, Bayona, diciembre de 2021, 513 Me gusta, 43 comentarios y 255 veces compartido)

### La vacunación como práctica preventiva para el cuidado de la salud

Aunque la vacunación (en tanto práctica preventiva) no se constituyó en un problema público (objeto de disputa y polémica) de peso en la arena público-mediática en el período estudiado, nuestra investigación tenía como propósito aportar al diseño de estrategias de comunicación de la política de inmunizaciones en un contexto de incremento de la desconfianza hacia la vacunación y la caída de las coberturas vacunales a nivel global. Por ello, como parte de la caracterización de la conversación pública decidimos examinar los argumentos circulantes (a favor y en contra) de la vacunación. Así, reconstruimos que el cuidado fue un eje transversal de la conversación en los momentos descritos, bajo el despliegue de la categoría *argumentos*, que incluyó enunciados favorables y contrarios a la vacunación covid-19, como práctica preventiva.

En la comunicación gubernamental, los argumentos a favor se apoyaron en el doble beneficio, individual y colectivo, y se presentaron sintetizados en eslóganes y/o *hashtag* en redes: “*Es bueno para vos, es bueno para todas y todos*”; “*Elegir vacunarse es cuidarse uno y cuidar al otro*”, acompañados de la frase “*Todxs somos protagonistas, vacúnate*”, invitando a formar parte de una gesta colectiva que requiere de una decisión y elección individual. La idea de cuidado fue recurrente: “*Sigamos cuidándonos y Argentina te cuida*”. En algunas publicaciones se enfatizó en el carácter igualador de las vacunas: “*Las vacunas nos igualan y son uno de los signos más robustos de equidad de un país*”, como parte de los valores de la campaña. Además de asociarse con el cuidado, se planteó su seguridad.

En *Página/12*, en línea con la comunicación gubernamental, se planteó el argumento del cuidado, enfatizando en la seguridad y efectividad, retomando fuentes científicas y del Ministerio de Salud, con referencia a la publicación de los resultados del ensayo clínico de fase 3 para Sputnik en *The Lancet*.

Validación crucial. La vacuna rusa Sputnik V tiene una eficacia superior al 91%, según publicó la prestigiosa revista *The Lancet*. El análisis de los ensayos clínicos fue validado por expertos independientes. No se registraron efectos adversos graves. (*Página/12*, febrero de 2021)

El énfasis en la idea de que vacunarse es cuidarse a uno mismo y al otro, y que las vacunas son seguras, respondieron a las dudas sobre la seguridad y el temor a los efectos adversos basados en la falta de evidencia científica, planteados tanto en la prensa digital como en las redes sociales. Referencias en medios como *La Nación* alimentaron la desconfianza hacia la vacuna, especialmente Sputnik V.

La primera condición para inocular una vacuna es la confianza con que cuenta entre los científicos y también en la gente común. Esa confianza no estaba. ¿Estará? Es probable que sí después de la publicación de *The Lancet*. Pero no se puede desconocer la audacia del gobierno local cuando comenzó una campaña de vacunación con un inmunizador del que se ignoraba todo. Tiró la moneda al aire. A cara o cruz. La apuesta de un ludópata. (*La Nación*, febrero de 2021)

La decisión individual de no vacunarse por la desconfianza hacia la vacuna también se escenificó en el mismo medio:

CD, de 45 años, comerciante [...] “Es una cuestión de libertades individuales. Yo decidí no vacunarme porque no tengo confianza en la vacuna. (*La Nación*, diciembre de 2021)

Si bien las dudas sobre la seguridad y la eficacia estuvieron presentes, excepcionalmente, en la prensa digital se observaron argumentos francamente en contra de la vacunación en general, pero con una mirada crítica y de distancia de los medios que las asociaron con miedos irracionales de la población.

La irrupción de la Sputnik V [...] en el escenario latinoamericano ha estado marcado por la polémica. Tras acordar el envío de 20 millones de dosis a Argentina [...] afloraron todo tipo de cuestionamientos: desde las dudas por la falta de información científica publicada en Occidente en el momento que se cerraron los primeros acuerdos y los dardos políticos para criticar la gestión de la pandemia, hasta los miedos y delirios que rayan las teorías de la conspiración. “Recuerden que lleva un chip comunista y castrochavista”, advirtió un usuario de Twitter. (*La Nación*, febrero de 2021)

En redes sociales, especialmente en Facebook, las publicaciones a favor pertenecieron a cuentas de sociedades científicas que también avalaron la seguridad y efectividad de las vacunas con información científica accesible, en línea con la comunicación gubernamental, respondiendo a dudas sobre la seguridad de vacunas desarrolladas y aprobadas en tiempo récord. El argumento vinculado con un acto de responsabilidad individual y colectiva apareció una vez que se superó la discusión sobre su efectividad y seguridad (“#lasvacunassonseguras”). En Twitter y YouTube el énfasis en la idea de que vacunarse es cuidarse y cuidarnos, y que las vacunas son seguras, respondieron a las dudas sobre la seguridad y temor sobre efectos adversos debido a su novedad y la posibilidad de no cumplir con fases de investigación habituales, apelando a la responsabilidad individual para asumir el riesgo.

Facebook fue la red social en la que más abiertamente se presentaron los argumentos en contra de la vacunación. Estos fueron presentados apelando a la ciencia, cuestionando la necesidad de la vacunación como respuesta a la pandemia, cultivando desconfianzas debido a su aprobación en emergencia y al desconocimiento de los riesgos y peligros, poniendo en duda su eficacia e inocuidad, en una asociación de la vacuna como negocio. Aunque poco numerosos, se registraron argumentos basados en teorías conspirativas que asociaron la vacunación con un escenario de complot a nivel nacional y/o global.

Si bien los cuestionamientos hacia la aplicación de la vacuna -basados en dudas sobre la seguridad y el temor a los efectos adversos- estuvieron presentes, las altas coberturas registradas de la vacuna covid-19 (96,5% de la cobertura de la población mayor de 18 años y un 89,4% de la cobertura total del país que incluye todas las edades, incluso los niños de 3 años y más)^32^ y la ausencia de posiciones explícitas y extendidas contra la vacunación, pueden leerse como indicadores del grado de aceptabilidad de las vacunas en general y la existencia de un mandato moral que responsabiliza a padres y madres sobre la vacunación de niños y niñas^8^. Más allá de este punto, cabe mencionar que una particularidad de Facebook, como red social, es la censura de algunos posteos que presentaban material sobre la vacunación contra el covid-19. A partir de la búsqueda realizada, se identificó el bloqueo sistemático de cuentas por parte de Facebook de aquellas personas usuarias que subieran materiales confusos, incompletos o falsos sobre la vacunación contra el covid-19. Aún con esta tendencia, fue posible identificar usuarios de esa red social con significativa actividad en el cuestionamiento de las vacunas contra el covid-19.

El término “antivacunas” y la referencia a grupos posicionados en contra de la vacunación nombrados y estudiados como grupos antivacunas^17^^,^^18^^,^^19^^,^^20^, no fue utilizado en publicaciones oficiales. Solo se los refirió implícitamente en entrevistas periodísticas concedidas por el presidente de la Nación en las que fue interrogado sobre esta cuestión. Desde su perspectiva, la actuación de grupos en contra de la vacunación se asociaba con la actuación irresponsable y oportunista de la oposición política y de los medios; con posturas terraplanistas o producto de una situación social de locura generalizada vinculada con la pandemia.

Significativamente, en la prensa digital tampoco se observaron referencias extendidas y explícitas que refirieran a movimientos, grupos o incluso comportamiento “antivacunas”. Y en redes sociales, especialmente en Twitter, la cita que mencionó de manera directa a la postura antivacunas fue irónica.

Qué rol pequeñito y humillante ser antivacunas. (Twitter, Angela Lerena, febrero de 2021, retuits 1.422- likes 12.105)

En YouTube tampoco se observaron vinculaciones directas a posturas antivacunas. En cualquier caso, la actitud reticente o antivacuna covid-19 fue descalificada y fueron asimiladas posiciones anticientíficas y/o conspirativas, provocando distancia en las personas que manifestaban temores y críticas basadas en dudas legítimas. No es casual que el término antivacunas tampoco sea un rótulo identificatorio aceptado, incluso en casos donde se presentaban actitudes reticentes a ciertas vacunas, enfocadas en la decisión individual de no aplicarla como práctica de cuidado (de los efectos adversos).

NP integra la red y vive en Córdoba con sus dos hijos, S., de 15 años con epilepsia, y A., de 8 años, con parálisis cerebral y síndrome de West. […] “Nos tratan de antivacunas cuando desde junio hemos rogado por una vacuna segura para los chicos y, acá, autorizaron el uso de una vacuna a puertas cerradas, donde todo es confidencial”, agregó Natalia. (*La Nación*, octubre de 2021)

### Los encuadres dominantes en la conversación sobre vacunas

**Figura 5:**
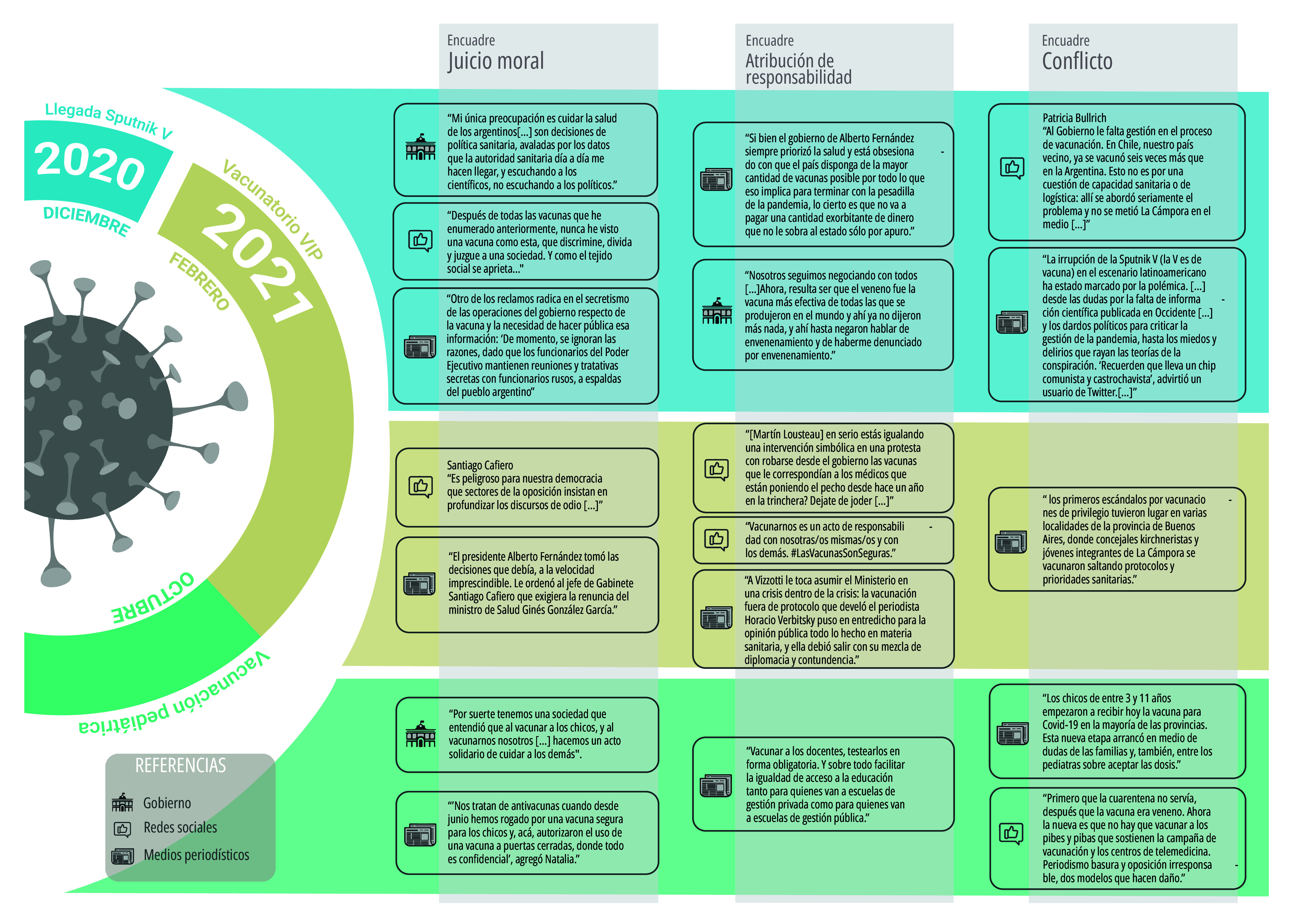
Fragmentos ilustrativos de los encuadres dominantes de la conversación pública sobre la vacunación y vacunas covid-19, por actor y/o plataforma, según tres eventos críticos (llegada de Sputnik V, “vacunatorio VIP” y vacunatorio pediátrica). Argentina, agosto 2020 - diciembre 2021.

Luego del análisis cualitativo temático, identificamos los encuadres de la conversación pública^26^^,^^27^^,^^28^ y las voces autorizadas sobresalientes para comprender el efecto de sentido de marcos e ideas potencialmente capaces de atribuir relevancia y promover una definición e interpretación, evaluación moral y/o recomendación de acción a propósito de las vacunas, en general, y en particular para el covid-19 (Figura 5).

Fuente: Elaboración propia.

El encuadre dominante del gobierno fue el del juicio moral, expresado en la priorización del cuidado de la salud y la vida, por sobre otros aspectos como la economía o la política, priorización respaldada en el consejo de expertos, que caracterizó la defensa de la vacunación como práctica de cuidado individual y colectiva en todo el período.

En la prensa digital y en las redes sociales, si bien el encuadre del juicio moral se mantuvo constante, en momentos críticos prevalecieron los encuadres de atribución de responsabilidades, señalando generalmente como responsable causal o político al gobierno nacional, independientemente del tono crítico o favorable; y el del conflicto en el contexto de una polarización ideológica del debate público. Respecto de este segundo encuadre, en ningún caso los conflictos refirieron a posiciones divergentes respecto de la práctica de la vacunación, sino que se vincularon con situaciones de conflicto que tuvieron como objeto las decisiones y prioridades de gestión gubernamental.

El caso de Facebook merece un tratamiento específico, ya que tanto la atribución de responsabilidades como el planteo de conflictos sí tuvieron como objeto a la vacunación. Concretamente, en algunas publicaciones, motivadas por intereses extrasanitarios, se atribuyó al gobierno la responsabilidad de promover vacunas innecesarias y riesgosas para la salud, pero también a la oposición. El conflicto surgió a propósito de miradas contrarias sobre la veracidad y la legitimidad de los argumentos esgrimidos para defender o cuestionar la vacunación y las vacunas, por lo general, formulados desde parámetros científicos allende las posiciones.

Más allá de las particularidades, en el conjunto de plataformas y actores analizados, el encuadre de juicio moral fue el dominante, como mostraron investigaciones precedentes^14^^,^^22^. En algunos casos, la vacunación contra el covid-19 se presentó como una responsabilidad individual y colectiva bajo el imperativo ético del cuidado de la vida; y la renuencia a la vacunación quedó justificada como medida de resguardo individual frente a los riesgos y efectos adversos de la vacunación y el ejercicio de la libertad de decidir sobre el propio cuerpo, la salud y el cuidado personal o de personas a cargo. El encuadre del juicio moral se advirtió en los términos en que fue valorada la ética de la función pública: valoración del rol del presidente Alberto Fernández como cuidador de la vida; sanción moral al gobierno por haber traicionado la confianza que el pueblo había depositado para el cuidado colectivo en ocasión del “vacunatorio VIP”; valoración por parte del gobierno de la generosidad rusa y la cooperación entre países para garantizar un acceso igualitario a la vacuna como bien público global.

Aunque la conversación adoptó en forma dominante el encuadre moral, como mostraron otras investigaciones^14^^,^^22^, también apeló a voces de autoridad científica, en particular de áreas como la infectología y la epidemiología. El discurso científico y sus reinterpretaciones se erigieron en el principal recurso para proveer ideas y argumentos sobre la seguridad y efectividad de la vacuna, ofreciendo datos y argumentos al discurso político, periodístico y público para disputar sentidos sobre aspectos del fenómeno en la arena pública bajo diferentes modalidades.

## DISCUSIONES Y CONCLUSIONES

La conversación pública en el periodo analizado se centralizó en el problema público de la gestión de la pandemia y en particular en la fabricación, adquisición y distribución de la vacuna covid-19 y su campaña de vacunación, frente a un tratamiento marginal de las vacunas del calendario. La vacunación contra el covid-19 y su campaña contaron con una muy alta aceptación social asociada al mandato moral del cuidado de sí y de otras personas^4^^,^^5^^,^^6^^,^^8^, evidenciada en una alta tasa de cobertura para toda la población en condiciones de vacunarse con base en los criterios de la autoridad sanitaria^32^.

El gobierno nacional se constituyó en propietario del problema, logrando imponer y estabilizar una narrativa que priorizó el argumento del cuidado de la salud y la vida, por sobre la economía o la política, y la vacunación como medida de cuidado individual y colectivo. Además, defendió la seguridad de la vacuna covid-19, y obvió mencionar o desestimó las actuaciones de los grupos reticentes o contrarios a la vacunación, salvo para identificarlos como parte de operaciones de desprestigio en el marco de disputas comerciales o actitudes malintencionadas, extemporáneas y/o irracionales. Para la estabilización del problema fue fundamental el “*standing*”^33^ de la autoridad científica, en particular, y como lo evidenciaron otras investigaciones sobre la pandemia^34^, en particular la de los expertos y las expertas en epidemiología e infectología y sus sociedades científicas.

En la conversación pública, la prensa digital en general convalidó el encuadre gubernamental, en línea con estudios precedentes^14^^,^^15^^,^^22^^,^^34^^,^^35^, que refieren a la correlación habitual entre los marcos (*frames*) de las fuentes oficiales y los periodísticos, salvo en coyunturas específicas en las que primó la atribución de responsabilidades al gobierno, en tanto responsable político de la pandemia en un contexto de polarización ideológica del debate político hacia el final del periodo analizado^14^^,^^22^.

Las posiciones críticas o contrarias a la vacunación contra el covid-19 fueron relativamente marginales y discurrieron mayoritariamente en redes sociales. Estas compartieron el encuadre moral dominante para criticar al gobierno, reavivaron y favorecieron la circulación de controversias preexistentes sobre la vacunación^4^^,^^5^^,^^8^ y pusieron en cuestión los modos en que el Estado gestionó y comunicó la vacunación. En Twitter y YouTube se registró una importante circulación de dudas sobre la seguridad de la vacuna, como también expresiones de temor por sus posibles efectos adversos, semejantes a posiciones reticentes relevadas en investigaciones previas^8^^,^^10^^,^^11^^,^^12^^,^^18^. En Facebook, aunque fue muy relevante el rol que tuvieron las sociedades científicas en el respaldo a la vacuna covid-19 y la campaña de vacunación, se registraron publicaciones declaradamente contrarias a su aplicación, que en ningún caso fueron suscritas por personas o grupos que se identificaron como “antivacunas”, a diferencia de lo que ocurre en otros países^19^.

Este espectro reticente amplio y heterogéneo comprende desde dudas sobre la seguridad y eficacia de las vacunas y temores comprensibles sobre posibles efectos adversos, hasta planteos seudocientíficos y/o conspirativos abiertamente contrarios a la vacunación^20^. Estas publicaciones reivindican derechos y libertades, adoptan frecuentemente una denominación y un lenguaje científicos, abrevan en voces autorizadas y fuentes biomédicas, y consideran formas de entender al cuidado asociadas con una “nueva conciencia de la salud”^36^, extendida en estratos medios urbanos que valoran la toma de decisiones informadas y autónomas como principal salvaguarda de la salud individual, en un contexto de creciente desconfianza de la población hacia las instituciones científicas y gubernamentales, encargadas de la salud pública^8^^,^^36^.

En la era del declive de la credibilidad de las instituciones históricamente reconocidas como productoras de información, conocimiento, verdad y políticas, como las instituciones mediáticas, científicas y médicas^8^^,^^11^^,^^19^, los estudios sobre la conversación pública en la web 2.0 ofrecen evidencia sobre la significación social de las vacunas y la vacunación, y sobre el espectro reticente a la vacunación que resultan indispensables para un replanteo de la política de comunicación de las inmunizaciones que comprenda estrategias diferenciadas que permeen conversaciones que discurren por diferentes comunidades y plataformas en el marco de sociedades hipermediatizadas^29^^,^^30^.

Concluimos que el estudio ofrece aportes para la política sanitaria en un contexto de caída de las coberturas vacunales, más allá de lo acontecido durante la pandemia por covid-19, indicando la necesidad de diversificar y segmentar mensajes, y recentrar la comunicación en todas las vacunas del calendario. Asimismo, el concepto de vacuna asociado con el cuidado individual y colectivo debe complejizarse para que contemple las nuevas formas de entender el cuidado de algunos sectores sociales, considerando dudas razonables y controversias legítimas, y respondiendo con evidencia porque, aun en contextos críticos, la ciencia, con las reinterpretaciones del conocimiento que produce, sigue siendo una voz autorizada en la modernidad tardía.
